# Early Methanogenic Colonisation in the Faeces of Meishan and Yorkshire Piglets as Determined by Pyrosequencing Analysis

**DOI:** 10.1155/2014/547908

**Published:** 2014-01-06

**Authors:** Yong Su, Gaorui Bian, Zhigang Zhu, Hauke Smidt, Weiyun Zhu

**Affiliations:** ^1^Laboratory of Gastrointestinal Microbiology, College of Animal Science and Technology, Nanjing Agricultural University, Nanjing 210095, China; ^2^Laboratory of Microbiology, Wageningen University, Dreijenplein 10, Wageningen 6703 HB, The Netherlands

## Abstract

Gut methanogenic archaea of monogastric animals are considered to be related to energy metabolism and adipose deposition of the host; however, information on their development in young piglets is limited. Thus, to investigate early methanogenic colonisation in the faeces of Meishan and Yorkshire piglets, faecal samples were collected from piglets at 1, 3, 7, and 14 days after birth and used to analyse the methanogenic community with 16S rRNA gene pyrosequencing. Results showed that the diversity of the methanogenic community in the faeces of neonatal piglets decreased from one to 14 days of age, as the total methanogen populations increased. The age of piglets, but not the breed, significantly affected the diversity of the methanogenic community which was dominated by the genus *Methanobrevibacter*. From the ages of one to 14 days, the abundance of *M. smithii*-related operational taxonomic units (OTUs) increased significantly, while the abundances of *M. thaueri*- and *M. millerae*-related OTUs decreased significantly. The substitution of *M. smithii* for *M. thaueri/M. millerae* was faster in Yorkshire piglets than in Meishan piglets. These results suggest that the early establishment of microbiota in neonatal piglets is accompanied by dramatic changes in the methanogenic community, and that the changes vary among pigs of different genotypes.

## 1. Introduction

Methanogenic archaea exist widely in the gastrointestinal (GI) tract of many vertebrates and invertebrates [1–3]. Methanogens can use hydrogen and other compounds such as formate, methanol, and acetate, as electron donors for the production of methane. Methane formation not only contributes to global warming as a greenhouse gas, but it also represents an energy loss for the animal [4]. Recently, methanogenic archaea in the gut of monogastric animals, including humans, have been studied intensively because gut methanogens are considered to be related to energy metabolism and adipose deposition of the host [5]. In addition, methane produced by methanogens might play an important role in the pathogenesis of several intestinal disorders, including colon cancer, inflammatory bowel disease, irritable bowel syndrome, and diverticulosis [6]. Thus far, only limited reports on gut methanogens in pigs have been available. Based on archaeal 16S ribosomal RNA (rRNA) gene clone library analysis, methanogens belonging to the genus *Methanobrevibacter *were found to be predominant in pig faeces [7, 8].

The GI microbiota of newborn animals play a fundamentally important role in the development of intestinal function and the innate immune system [9–12]. The infant gut ecosystem undergoes a dramatic transition from an essentially sterile state to extremely dense colonisation, ending with the establishment of an adult-like microbial community [13, 14]. In contrast to the gut microbiota of adult animals, the microbiota of neonates are more variable and less stable over time. The fragile ecological system is not only a disease risk to the newborn gut, but it can also have a long-term effect on it's later life health [15–17]. Comparing the gut bacterial communities of neonatal animals and humans which have been intensively studied, information on gut methanogenic communities of neonatal monogastric animals such as pigs is still limited.

The Meishan and Yorkshire breeds are typical obese and lean pigs, respectively; thus, their energy metabolism might be distinctive. It has been found that obese Meishan pigs harbour relatively higher numbers of *Firmicutes* and lower numbers of *Bacteroidetes* compared to lean breeds [18]. Moreover, a recent study showed that lean Landrace pigs harboured a greater diversity of methanogens and a higher number of methanogen *mcrA *gene copies than the obese Erhualian pigs [8]. However, it is not clear whether the different composition of gut methanogens in the various pig breeds is related to the early methanogenic colonisation of newborn piglets. Therefore, the aim of this study was to investigate the development of methanogenic archaea in the faeces of newborn Meishan and Yorkshire piglets by using high throughput pyrosequencing analysis of PCR-amplified 16S rRNA genes.

## 2. Materials and Methods

### 2.1. Collection of Faecal Samples

This study was approved by the Nanjing Agricultural University Animal Care and Use Committee. All of the Meishan and Yorkshire pigs were raised on a commercial farm in Jiangsu province, China. Candidate sows with a similar expected delivery date were chosen from both breeds and injected intramuscularly with cloprostenol (0.2 mg per sow) at 10:00 am on day 113 of pregnancy to ensure homochronous deliveries. Four vaginally delivered litters of piglets (each litter with 10–12 piglets) for each pig breed which delivered homochronously within two hours were finally used in this study. The same diets were formulated for Meishan and Yorkshire sows according to nutrient requirements of the National Research Council. Fresh faeces were collected from the piglets at 1, 3, 7 and 14 days of age and immediately stored at −28°C for further molecular analysis.

### 2.2. DNA Extraction and PCR Amplification

Total genomic DNA was isolated from the faecal samples using a commercially available stool DNA extraction kit, according to the instructions of the manufacturer (QIAamp DNA Stool Mini Kit: Qiagen, Hilden, Germany). The concentration of the extracted DNA was determined using a NanoDrop 1000 spectrophotometer (Thermo Scientific Inc., Wilmington, DE, USA).

To analyse the taxonomic composition of the methanogenic community, *Archaea*-specific primers (Arch344F 5′-ACG GGG YGC AGC AGG CGC GA-3′ and Arch915R 5′-GTG CTC CCC CGC CAA TTC CT-3′) targeting the V3–V6 region of the 16S rRNA gene were chosen for the amplification and subsequent pyrosequencing of the PCR products [19, 20]. The PCRs were carried out in triplicate in 50 µL reactions with 10 µL 5-fold reaction buffer, 50 ng of DNA, 0.4 mM of each primer, 0.5 U Pfu polymerase (TransStart-FastPfu DNA Polymerase, TransGen Biotech), and 2.5 mM dNTPs. The amplification program consisted of an initial denaturation step at 95°C for 2 min. This was followed by 30 cycles, where one cycle consisted of 95°C for 30 s (denaturation), 58°C for 90 s (annealing), 72°C for 30 s (extension), and a final extension of 72°C for 5 min. PCR products were visualised on agarose gels (2% in TBE buffer) containing ethidium bromide, and purified with a DNA gel extraction kit (Axygen, China).

### 2.3. Pyrosequencing and Bioinformatics

Prior to sequencing, the DNA concentration of each PCR product was determined using a Quant-iT PicoGreen double-stranded DNA assay (Invitrogen, Germany) and was quality-controlled on an Agilent 2100 Bioanalyzer (Agilent, USA). Amplicon pyrosequencing was performed from the A end using a 454/Roche A sequencing primer kit on a Roche Genome Sequencer GS-FLX Titanium platform at Majorbio Bio-Pharm Technology Co., Ltd., Shanghai, China.

PCR-amplified fragments were blunted and tagged on both ends with ligation adaptors that contained a unique 10 bp sequence (sample specific barcode sequence) and a short 4-nucleotide sequence (TCAG) called sequencing key, which were recognised by the system software and the priming sequences. All pyrosequencing reads were binned according to barcode and primer sequences. The resulting sequences were further screened and filtered for quality. Sequences that were shorter than 200 bp in length, contained ambiguous characters, contained over two mismatches to the primers, or contained mononucleotide repeats of over six nt were removed. To assess bacterial diversity among samples in a comparable manner, a randomly selected, 2564-sequence (the lowest number of sequences in the 32 samples) subset from each sample was aligned using the “align.seqs” command and compared with the SILVA archaeal database (SILVA version 108). The aligned sequences were further trimmed and the redundant reads were eliminated using successively the “screen.seqs,” “filter.seqs,” and “unique.seqs” commands. The “chimera.slayer” command was used to determine chimeric sequences. The “dist.seqs” command was performed, and unique sequences were clustered into operational taxonomic units (OTUs) defined by 97% similarity [21] using CD-HIT-OUT program [22]. We also calculated the coverage percentage using Good's method [23], abundance-based coverage estimator (ACE), bias-corrected Chao richness estimator, and the Shannon and Simpson diversity indices. A heat map was generated using custom Perl scripts. All the analyses were performed using the MOTHUR program (http://www.mothur.org/) [24]. Principal coordinate analysis (PCoA) was conducted based on the weighted UniFrac distance [25].

### 2.4. Phylogenetic Analysis

Sequences of OTUs with an abundance higher than 0.1% with total reads were derived and used for construction of a phylogenetic tree. Homology searches of the GenBank DNA database were further performed with a BLAST search. Sequences of OTUs-related species were retrieved from the GenBank database. Multiple sequence alignments were performed using ClustalX1.81 [26]. Phylogenetic analysis was performed with the MEGA 3.1 software package. An unrooted phylogenetic tree was constructed using the neighbour-joining method [27].

### 2.5. Real-Time PCR Quantification of Methanogenic Archaea

Quantitative PCR was performed on an Applied Biosystems 7300 Real-Time PCR System (ABI) using SYBR Green as the fluorescent dye. The reaction mixture (25 *μ*L) consisted of 12.5 *μ*L of IQ SYBR Green Supermix (Bio-Rad), 0.2 *μ*M of each primer set, and 5 *μ*L of the template DNA. The amount of DNA in each sample was determined in triplicate, and the mean values were calculated. *Archaea*-specific primers Arch 344 f [19]) and Arch806 [28] were used to quantify the 16S rRNA gene of archaeal methanogens under the following conditions: an initial DNA denaturation step at 95°C for 10 min, followed by 40 cycles of denaturation at 95°C for 15 s, and primer annealing and extension at 60°C for 1 min. DNA from cells of a pure culture of *M*. *smithii* was used as the standard. Results are expressed as the numbers of 16 S rRNA gene copies per gram of faeces.

### 2.6. Statistical Analysis

The effects of pig breed and age on the composition of the methanogenic archaeal community were tested for significance using a two-way analysis of variance (ANOVA) program in the Statistical Package for the Social Sciences (SPSS17.0). Significant differences were declared when *P* < 0.05.

## 3. Results

### 3.1. Metrics of Pyrosequencing Analysis

**Figure 1:**
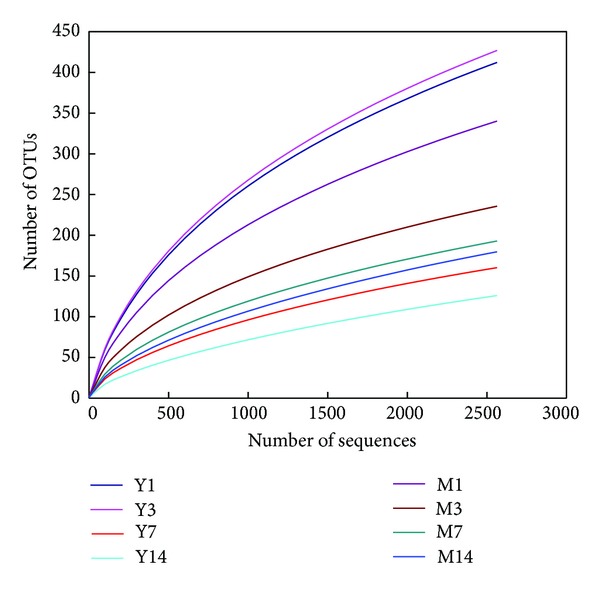
Rarefaction curves comparing the number of reads with the number of phylotypes (OTUs) found in the 16S rRNA gene libraries from faecal methanogens of Meishan and Yorkshire piglets. M: Meishan piglets; Y: Yorkshire piglets; 1, 3, 7, and 14 represent the ages of 1, 3, 7, and 14 days.

Across all 32 samples, 132 138 quality-trimmed sequences from a total of 165 338 reads were classified as* Archaea*. The average length of the quality-trimmed sequences was 483 bp. The rarefaction curves generated by MOTHUR plotting the number of reads against the number of OTUs indicated that using 2564 reads per sample (the minimum number of sequences passing all quality control measures across all samples) for the final analysis was adequate as the curves tended to approach the saturation plateau (Figure 1).

### 3.2. Diversity

Coverage, number of OTUs, and statistical estimates of species richness for each group at a genetic distance of 3% are presented in Table 1. The age of the piglets significantly affected the diversity indices (Shannon and Simpson) and richness estimators (ACE and Chao) of faecal methanogenic archaeal community (*P* < 0.05); there was no significant difference between the pig breeds. In both breeds, the piglets harboured a higher diversity of faecal methanogens at 1 and 3 days of age than at 7 and 14 days (*P* < 0.05).

### 3.3. Taxonomic Composition

Across all reads, 99.91% were identified as class* Methanobacteria*, while *Thermoplasmatales* composed the remaining 0.09%. Within class *Methanobacteria*, family Methanobacteriaceae was predominant, represented by genera *Methanobrevibacter* and *Methanosphaera*. A very high abundance of genus *Methanobrevibacter *(95.01%–100%) was found in all samples; thus, further analysis was performed at the species (OTU) level.

**Figure 2:**
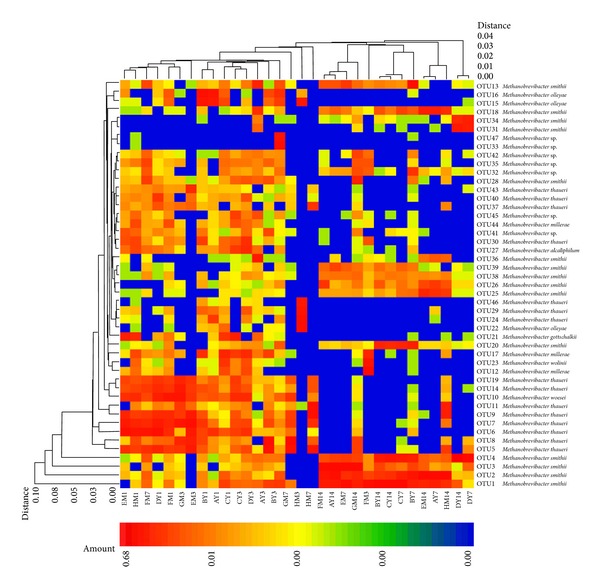
Double dendrogram showing the distribution of methanogens in the faeces of Meishan and Yorkshire piglets (OTU level). The relationship among samples was determined using Bray distance and the complete clustering method. A total of 47 OTUs with an abundance higher than 0.3% within total methanogens were selected for the analysis. The heatmap plot depicts the relative abundance of each OTU (variables clustering on the *y*-axis) within each sample (*x*-axis clustering). The relative values for the OTUs are depicted by colour intensity in the legend at the top of the figure. Clusters based on the distance of all samples along the *x*-axis and the different OTUs along the *y*-axis are indicated at the top and left of the figure, respectively. A–D: litter numbers of Yorkshire piglets; E–H: litter numbers of Meishan piglets.

**Figure 3:**
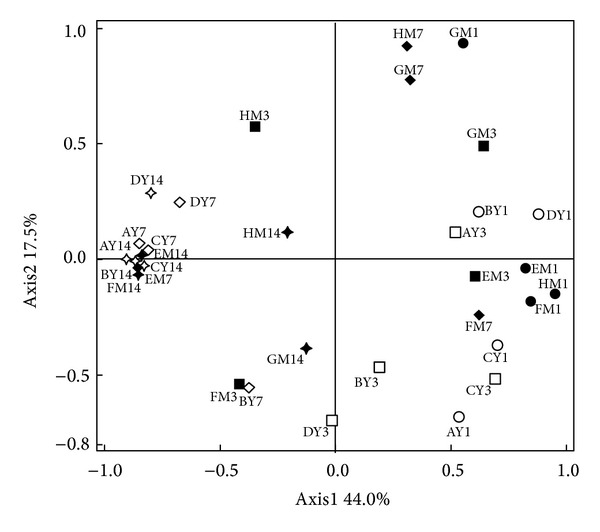
Principal coordinates analysis of weighted UniFrac values in the fecal methanogens of Meishan and Yorkshire piglets. Sample identifiers are the same as in Figures 1 and 2.

Clustered heat map analysis based on the archaeal community profiles at the OTU level showed that most samples taken from the piglets at 1 and 3 days of age were grouped together and separated from the samples taken at 7 and 14 days (Figure 2). In addition, PCoA analysis also showed that the first principal coordinate (P1), which explains 44.0% of the variation, separated the archaeal communities of most of the piglets at 7 and 14 days from the samples of younger animals (Figure 3).

**Figure 4:**
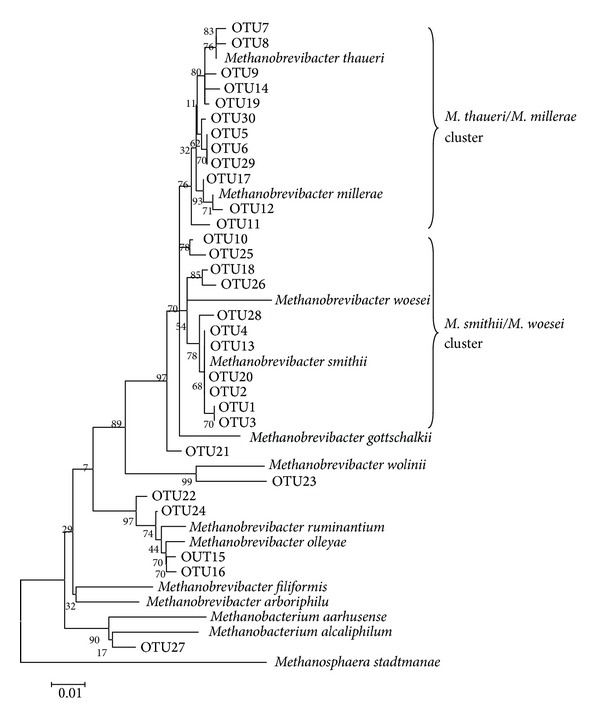
Unrooted phylogenetic tree of *Methanobrevibacter* spp. reference strains and predominant OTUs in the libraries from faecal methanogens of Meishan and Yorkshire piglets. The reference bar indicates 1% sequence dissimilarity.

Sequences of predominant OTUs with a relative abundance higher than 0.5% of total reads and reference sequences of *Methanobrevibacter* spp. were used for construction of the phylogenetic tree. All of the OTUs were closely related to genus *Methanobrevibacter *(Figure 4). Most of the OTUs were divided into two clusters—*M. smithii/M*.* woesei* cluster and *M*.* thaueri/M*. *millerae* cluster. OTUs 1, 2, 3, 4, 13, 20, and 28 were most closely related to *M. smithii*, whereas OTUs 5, 6, 7, 8, 9, 14, 19, 29, and 30 were most closely related to *M*.* thaueri*. OTUs with a relative abundance higher than 0.1% of total reads were further used for a significance test of relative abundance among different groups (Table 2). OTUs significantly affected by pig breed or age were mainly related to *M. smithii*,* M*.* thaueri*, and *M*. *millerae *(*P* < 0.05). Pig breed significantly affected the relative abundances of *M. smithii-* and *M*.* thaueri-*related OTUs, and pig age significantly affected the relative abundances of predominant *M. smithii*-, *M*.* thaueri*-, and* M*. *millerae*-related OTUs (*P* < 0.05) (Table 3). Interestingly, it was observed that the substitution of *M. smithii* for *M*.* thaueri/M*. *millerae* occurred faster in Yorkshire piglets than in Meishan piglets. Sequences most closely related to *M. smithii* became predominant at the age of 7 days in Yorkshire piglets, whereas this species dominated in the Meishan piglets only from day 14. In both breeds, from day 1 to day 14, the relative abundance of *M. smithii-*related OTUs increased significantly in the faeces, while the relative abundances of *M*.* thaueri-* and* M*. *millerae-*related OTUs decreased significantly (*P* < 0.05).

### 3.4. Quantification of 16S rRNA Gene Copies of Methanogenic Archaea

**Figure 5:**
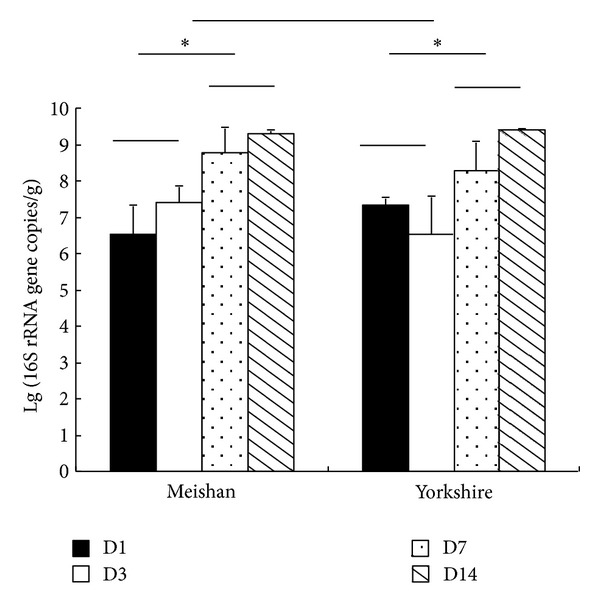
Real-time PCR quantification of 16S rRNA gene copies of methanogenic archaea in the faeces of Meishan and Yorkshire piglets. *P < 0.05, *n* = 4.

The absolute numbers of the 16S rRNA gene copies of methanogenic archaea in the faeces of the piglets were determined with real-time PCR assays (Figure 5). Pig breed did not affect faecal methanogen populations; however, in both breeds, significant differences were found among samples taken from the piglets at different ages. The numbers of faecal methanogen 16S rRNA gene copies found in the piglets at 7 and 14 days were significantly higher than those found at 1 and 3 days (*P* < 0.05).

## 4. Discussion

Since Ley et al. found that gut microbiota were associated with the energy metabolism of the host, the role of gut microbiota in the metabolism of the host has received more attention [29]. While numerous studies have focused on two major bacterial groups (*Firmicutes* and *Bacteroidetes*) in the gut of animals, it was shown in a germ-free mouse model that methanogens also play an important role in energy metabolism and adipose deposition [30]. Unlike the potential roles of methanogens in a host's energy metabolism and adipose deposition, the diversity and structure of methanogenic communities in pig gut have not been well understood.

Studies using 16S rRNA gene-based techniques indicate that the predominant species in ruminants belong to the genus* Methanobrevibacter* [31, 32]. Recently, a methanogenic archaeal 16S rRNA gene library of pig faeces was constructed wherein the clones were mainly separated into three clusters—*Methanobrevibacter*, *Methanosphaera*, and a group of uncultivated archaea [7]. In our previous study, the genus *Methanobrevibacter* was also found to dominate in the faeces of Erhualian and Landrace pigs [8], which is consistent with the result of the present study on Yorkshire and Meishan piglets. However, methanogenic genera besides *Methanobrevibacter* were seldomly detected in the present study, which suggests that the colonisation of other methanogens might occur later than the colonization of *Methanobrevibacter* spp.

To date, the diversity of methanogens in the human gut has been thought to be limited to several species among which *M. smithii *has been regarded as the main methane producer [33]. Furthermore, it has been shown that *M. smithii *concentration was higher in anorexic patients than in a lean population [34]. It was also reported that the gut microbiota from obese individuals were depleted in *M. smithii* [35]. These results indicate that *M. smithii* might play an important role in host energy metabolism. Similarly, it was observed that the abundance of *M. smithii-*related OTUs within the methanogenic 16S rRNA gene library was significantly higher in lean Landrace pigs than in obese Erhualian pigs [8]. In the present study, we found that *M. smithii* in the faeces of both breeds gradually became predominant during the first two weeks after birth. Furthermore, the dominance of *M. smithii* in the lean Yorkshire piglets occurred earlier than in obese Meishan piglets, which confirms the previous finding that lean animals and humans may harbour more *M. smithii* in their gut than obese ones.

The composition and diversity of neonatal microbiota are variable and vulnerable during early life until a stable ecosystem is established. Facultative anaerobes such as *Enterobacteriaceae, *enterococci, streptococci, and staphylococci, have been reported to be the predominant intestinal microbiota in infants during the first week [36, 37], as they can survive in the oxygen-containing gut. As oxygen is comsumed, anaerobic microorganisms, such as *Ruminococcus, Bacteroides*, and* Bifidobacterium*, gradually thrive [14, 38, 39]. An increasing diversity in the bacterial community was also observed during the development of gut microbiota in newborn piglets [40]. In contrast to the bacterial community, we found that the diversity indices of the faecal methanogenic community of Meishan and Yorkshire piglets decreased from 1 day to 14 days, which suggests that the colonisation of gut methanogens in neonatal piglets is the result of mutual selection between the methanogens and the host. This result is also consistent with the previous finding that the diversity of methanogens in the human and monogastric animal gut has been thought to be limited to several species.

In the present study, although the diversity of the methanogen populations decreased with the age of the newborn piglets, the numbers of methanogens increased significantly and reached 10^9^ copies of 16S rRNA gene per gram of faeces, which is similar to values observed for faeces of grown pigs and suckling piglets before weaning [8]. Combined with the results of diversity and taxonomic composition, this result suggests that a stable methanogenic community is established in the gut of piglets at the age of 14 days.

Interestingly, we found that from day 1 to day 14, the abundance of *M. smithii-*related OTUs in the faeces increased significantly, while the abundances of *M*.* thaueri-* and* M*. *millerae-*related OTUs decreased significantly. It was found that *M*.* thaueri-* and* M*. *millerae-*related OTUs dominated in the methanogenic 16 S rRNA gene library from the faeces of the Meishan and Yorkshire piglets three days after birth. Although these two species could be found in the hindgut of pigs [8, 41], *M. smithii* was the most abundant species in previous studies [7, 8]. In the gut, *M. smithii* converts H_2_, CO_2_, and formate into CH_4_ using carbon as the terminal electron acceptor. In contrast, *M. thaueri* only grows and produces methane from H_2_ and CO_2_ and does not grow or produce methane from formate, acetate methanol, trimethylamine, or methanol with H_2_ [41]. Considering the potential role of *M. smithii* in energy metabolism, this result indicates that the substitution of *M. smithii* for other *Methanobrevibacter* spp. might be important to the gut microbial ecosystem. We found that this substitution occurred more quickly in the Yorkshire piglets than in the Meishan piglets. Further studies are still needed to understand whether this difference is related to the different phenotype of these two breeds.

The microbiota of newborns mainly travel from the mother's vagina, skin, and faeces [42, 43]. In addition, diet composition is regarded as the main factor affecting gut microbiota. It was reported that Erhualian (similar breed to Meishan) sows had higher concentrations of milk lactose and fat but lower concentration of milk protein as compared to traditional Western breeds [44], which may be a possible reason for the existence of *M*.* thaueri* and* M*. *millerae* in the faeces of newborn piglets in relatively high abundance. Further studies are needed to reveal their potential role in the gut of newborn piglets.

## 5. Conclusions

In conclusion, the present study showed that the methanogenic community in the faeces of Meishan and Yorkshire neonatal piglets was dominated by members of the genus *Methanobrevibacter*, represented by *M. smithii*,* M*.* thaueri*, and* M*. *millerae*. The structure of the methanogenic community was significantly affected by the age of piglets; however, pig breed could also affect the substitution of different *Methanobrevibacter *spp.

## Figures and Tables

**Table 1:** Phylotype coverage and diversity estimation of the 16S rRNA gene libraries from faecal methanogens of Meishan and Yorkshire piglets^1^.

Breed	Age (d)	OTUs	ACE	Chao	Shannon	Simpson	Coverage
Meishan	1	340.3	697.1	564.2	4.263	0.045	0.938
	3	235.8	484.0	398.3	3.551	0.085	0.958
	7	193.0	470.9	343.1	2.797	0.207	0.963
	14	179.8	502.4	353.9	2.505	0.279	0.963

Yorkshire	1	412.3	783.4	658.4	4.732	0.029	0.928
	3	427.0	791.1	689.4	4.789	0.028	0.924
	7	160.3	416.8	277.8	2.311	0.326	0.968
	14	126.0	434.9	272.0	1.764	0.419	0.972

SEM^2^		137.7	250.95	214.0	1.297	0.168	0.023

Effect (*P* value)	Breed	0.184	0.409	0.307	0.664	0.185	0.236
	Age	0.000	0.048	0.001	0.000	0.000	0.001
	Breed × age	0.049	0.346	0.102	0.063	0.138	0.092

^1^The operational taxonomic units (OTUs) were defined with 3% dissimilarity. The coverage percentages, richness estimators (ACE and Chao), and diversity indices (Shannon and Simpson) were calculated.

^2^SEM: standard error of means, *n* = 4.

**Table 2:** Relative abundances of predominant OTUs (percentage) in the 16S rRNA gene libraries from faecal methanogens of Meishan and Yorkshire piglets^1^.

OTUs	Yorkshire				Meishan				SEM^2^	Effect (*P* value)			Closest reference strain
	D1	D3	D7	D14	D1	D3	D7	D14		Breed	Age	Breed × age	
OTU2	0.53	0.69	20.63	5.79	0.20	0.83	1.39	32.0	17.0	0.741	0.046	0.034	*M. smithii *
OTU6	6.89	1.20	0.09	0.00	10.16	0.68	3.41	0.08	5.07	0.293	0.001	0.679	*M. thaueri *
OTU7	3.19	1.31	0.03	0.00	9.87	3.92	2.97	0.26	4.50	0.024	0.012	0.392	*M. thaueri *
OTU10	3.64	1.57	0.02	0.00	6.14	3.22	1.60	0.10	3.23	0.157	0.011	0.860	*M. woesei *
OTU14	2.26	0.60	0.02	0.00	2.80	2.38	1.33	0.14	1.78	0.108	0.028	0.731	*M. thaueri *
OTU17	2.56	2.22	0.01	0.01	0.74	2.39	0.17	0.16	1.76	0.547	0.017	0.508	*M. millerae *
OTU19	2.12	0.45	0.00	0.00	2.52	1.51	1.19	0.11	1.37	0.097	0.004	0.738	*M. thaueri *
OTU20	0.20	0.64	3.26	2.57	0.20	0.03	0.12	0.22	1.88	0.015	0.204	0.217	*M. smithii *
OTU21	2.89	0.35	0.02	0.00	3.23	0.03	0.00	0.01	1.98	0.997	0.002	0.984	*M. gottschalkii *
OTU26	0.10	0.25	1.21	0.61	0.05	0.22	0.13	2.00	0.88	0.812	0.005	0.010	*M. smithii *
OTU27	1.49	1.49	0.08	0.01	0.98	0.19	0.24	0.02	1.03	0.223	0.049	0.403	*M. alcaliphilum *
OTU30	0.67	1.09	0.03	0.00	1.15	1.21	0.14	0.03	0.93	0.560	0.032	0.956	*M. thaueri *
OTU38	0.08	0.34	0.53	0.66	0.12	0.17	0.40	0.86	0.36	0.864	0.001	0.542	*M. smithii *
OTU39	0.03	0.19	0.55	1.00	0.03	0.12	0.41	0.52	0.53	0.324	0.026	0.756	*M. smithii *
OTU40	1.21	0.54	0.02	0.00	0.75	0.04	0.22	0.02	0.69	0.409	0.019	0.594	*M. thaueri *
OTU44	0.66	0.49	0.02	0.00	1.06	0.20	0.11	0.02	0.55	0.750	0.005	0.544	*M. millerae *
OTU48	0.15	0.34	0.53	0.40	0.06	0.09	0.16	0.61	0.28	0.134	0.010	0.083	*M. millerae *
OTU52	0.56	0.05	0.01	0.00	1.00	0.40	0.19	0.02	0.53	0.139	0.012	0.796	*M. thaueri *
OTU63	0.42	0.20	0.01	0.00	0.80	0.17	0.09	0.01	0.30	0.077	0.000	0.088	*M. thaueri *
OTU69	0.59	0.20	0.02	0.00	0.51	0.06	0.20	0.01	0.27	0.875	0.000	0.299	*M. thaueri *
OTU71	0.26	0.31	0.00	0.00	0.68	0.14	0.14	0.01	0.31	0.285	0.005	0.146	*M. millerae *
OTU73	0.08	0.13	0.60	0.35	0.01	0.08	0.03	0.25	0.27	0.016	0.038	0.067	*M. smithii *
OTU74	0.41	0.63	0.00	0.00	0.08	0.35	0.00	0.00	0.40	0.243	0.035	0.697	*M. olleyae *
OTU77	1.00	0.03	0.02	0.00	0.36	0.01	0.00	0.00	0.48	0.219	0.003	0.278	*M. millerae *
OTU78	0.38	0.15	0.02	0.00	0.51	0.09	0.20	0.02	0.23	0.263	0.000	0.487	*M. thaueri *
OTU82	0.28	0.14	0.01	0.00	0.33	0.07	0.40	0.05	0.21	0.075	0.010	0.047	*M. thaueri *
OTU85	0.25	0.13	0.00	0.00	0.39	0.35	0.10	0.02	0.26	0.175	0.047	0.862	*M. thaueri *
OTU86	0.17	0.16	0.02	0.00	0.44	0.16	0.29	0.00	0.20	0.024	0.010	0.153	*M. thaueri *
OTU88	0.33	0.04	0.00	0.00	0.49	0.20	0.12	0.00	0.25	0.143	0.002	0.845	*M. thaueri *
OTU91	0.67	0.07	0.00	0.00	0.33	0.00	0.04	0.01	0.35	0.399	0.006	0.572	*M. millerae *
OTU93	0.51	0.24	0.00	0.00	0.18	0.17	0.00	0.00	0.29	0.291	0.045	0.570	*M. millerae *
OTU103	0.04	0.04	0.17	0.32	0.02	0.04	0.10	0.21	0.15	0.277	0.002	0.821	*M. smithii *
OTU104	0.26	0.10	0.00	0.00	0.24	0.22	0.10	0.00	0.17	0.338	0.010	0.706	*M. thaueri *
OTU107	0.16	0.06	0.00	0.00	0.47	0.05	0.14	0.00	0.18	0.007	0.000	0.017	*M. thaueri *
OTU109	0.16	0.38	0.03	0.00	0.20	0.04	0.03	0.02	0.19	0.217	0.038	0.073	*M. millerae *
OTU110	0.22	0.18	0.04	0.00	0.27	0.08	0.04	0.02	0.17	0.892	0.018	0.786	*M. millerae *

^1^OTUs: the abundances that were significantly affected by pig breed, age, or the interaction between breed and age are shown in this table.

^2^SEM: standard error of means, *n* = 4.

**Table 3:** Relative abundances (percentage) of *M. smithii-*, *M. thaueri-*, *M. millerae-*, and *M. olleyae*-related OTUs in the 16S rRNA gene libraries from faecal methanogens of Meishan and Yorkshire piglets.

Breed	Age (d)	Relative abundance within total methanogens			
		*M. smithii-*related OTUs	*M. thaueri-*related OTUs	*M. millerae-*related OTUs	*M. olleyae-*related OTU
Meishan	1	1.81	61.11	18.43	1.67
	3	8.13	26.79	23.68	26.25
	7	26.28	57.54	7.29	0.000
	14	78.51	7.52	6.99	0.000

Yorkshire	1	3.68	33.01	27.72	10.25
	3	16.89	18.95	24.87	5.35
	7	88.41	0.79	3.74	0.13
	14	94.27	0.02	0.85	0.000

SEM^1^		41.67	31.78	16.06	14.53

Effect (*P* value)	Breed	0.003	0.010	0.969	0.521
	Age	0.000	0.020	0.009	0.081
	Breed × age	0.017	0.198	0.715	0.173

^1^SEM: standard error of means, *n* = 4.
